# Supplementary material for: Autoimmune responses to myelin-associated proteins as diagnostic and prognostic biomarkers of relapsing-remitting multiple sclerosis: Associations with human herpesvirus-6 and Epstein-Barr virus reactivation
Source: J Adv Res. 2025 Feb 16;78:543–53. doi: 10.1016/j.jare.2025.02.021 (PMC12684960; doi:10.1016/j.jare.2025.02.021)
Supplement: Supplementary Data 1 [file mmc1.docx]

**ELECTRONIC SUPPLEMENTARY FILE (ESF)**

**Autoimmune responses to myelin-associated proteins as diagnostic and prognostic biomarkers of relapsing-remitting multiple sclerosis: associations with human herpesvirus-6 and Epstein-Barr Virus reactivation.**

Aristo Vojdani^1,2 *^, Abbas F. Almulla^3,4,5,6**^, Elroy Vojdani^7^, Jing Li ^3,4^, Yingqian Zhang ^3,4^ ^**^, Michael Maes^3-5,8-11 **^

*Co-joint first authors; ** Co-corresponding authors.

^1^Immunosciences Lab, Inc., Los Angeles, CA 90035, USA.

^2^Cyrex Laboratories, LLC, Phoenix, AZ 85034, USA.

^3^Sichuan Provincial Center for Mental Health, Sichuan Provincial People’s Hospital, School of Medicine, University of Electronic Science and Technology of China, Chengdu 610072, China.

^4^Key Laboratory of Psychosomatic Medicine, Chinese Academy of Medical Sciences, Chengdu, 610072, China.

^5^Department of Psychiatry, Faculty of Medicine, Chulalongkorn University, and King Chulalongkorn Memorial Hospital, the Thai Red Cross Society, Bangkok, Thailand.

^6^Medical Laboratory Technology Department, College of Medical Technology, The Islamic University, Najaf, Iraq.

^7^Regenera Medical, Los Angeles, CA, USA

^8^Research and Innovation Program for the Development of MU - PLOVDIV– (SRIPD-MUP), Creation of a network of research higher schools, National plan for recovery and sustainability, European Union – NextGenerationEU.

^9^Department of Psychiatry, Medical University of Plovdiv, Plovdiv, Bulgaria, EU

^10^Research Center, Medical University of Plovdiv, Plovdiv, Bulgaria, EU

^11^Kyung Hee University, 26 Kyungheedae-ro, Dongdaemun-gu, Seoul 02447, Korea.

**ESF, Table 1. Excluded comorbidities in relapsing-remitting multiple sclerosis (RRMS) patients and healthy controls (HC).**

| **Type of disease** | **Name of disease** | **Notes** |
| --- | --- | --- |
| Psychiatric and neuropsychiatric disorders | Major depressive disorder (MDD) |  |
|  | Generalized anxiety disorder |  |
|  | Obsessive-compulsive disorder |  |
|  | Panic disorder |  |
|  | Other psycho-organic conditions unrelated to MS |  |
|  | Schizophrenia |  |
|  | Post-traumatic stress disorder |  |
|  | Substance use disorders | Except for nicotine dependance |
|  | Bipolar disorder |  |
|  | Chronic fatigue syndrome/myalgic encephalomyelitis (CFS/ME) |  |
| Neuroimmune Disorders | Chronic fatigue syndrome/myalgic encephalomyelitis (CFS/ME) |  |
| Neurodegenerative diseases | Parkinson disease |  |
|  | Alzheimer's disease |  |
| Neurodevelopmental disorder | Autism spectrum disorders |  |
| Autoimmune diseases | Type 1 diabetes mellitus |  |
|  | Rheumatoid arthritis |  |
|  | Inflammatory bowel disease |  |
|  | Lupus erythematosus |  |
|  | Psoriasis |  |
| Cancer | All known |  |
| Thyroid disorders | All known |  |
| Renal or liver disease | All known |  |
| Cardiovascular diseases | All known |  |
| Respiratory disease | Chronic obstructive pulmonary disease |  |

**ESF, Table 2. Sociodemographic and clinical data in patients with relapsing-remitting multiple sclerosis (RRMS) and healthy controls (HC).**

| **Variables** | **Healthy Controls (n=63)** | **RRMS Patients (n=55)** | **F/χ^2^** | **df** | **p-value** |
| --- | --- | --- | --- | --- | --- |
| Age (years) | 31.4 (7.1) | 29.5 (6.3) | 2.18 | 1/116 | 0.142 |
| Sex (M/F) | 33/30 | 37/18 | 2.70 | 1 | 0.133 |
| BMI (kg/m2) | 26.33 (4.50) | 24.94 (3.52) | 3.41 | 1/116 | 0.068 |
| Education (years) | 17.2 (5.1) | 18.4 (3.4) | 2.04 | 1/116 | 0.155 |
| Married/Separated | 26/37 | 31/24 | 2.68 | 1 | 0.139 |
| Smoking (N/Y) | 44/19 | 50/5 | 8.04 | 1 | 0.006 |
| Employment (N/Y) | 2/28 | 46/9 | 46.78 | 1 | <0.001 |
| MSSS | 0 | 1.71 (1.20) | MWU | - | <0.001 |
| EDSS | 0 | 1.03 (0.11) | MWU | - | <0.001 |
| PC-Severity | -0.921(0) | 1.05(0.198) | MWU | 1/116 | <0.001 |
| PC_3dUTPase-EBV | -0.662(0.745) | 0.759(0.655) | 119.51 | 1/116 | <0.001 |
| PC_3dUTPase-HHV-6 | -0.697(0.694) | 0.798(0.628) | 148.71 | 1/116 | <0.001 |
| PC_3EBNA | -0.579(0.810) | 0.664(0.755) | 73.71 | 1/116 | <0.001 |

M: Male, F: Female, kg: Kilogram, m2: squared meter, N: No, Y: Yes, MSSS: Multiple Sclerosis Severity Score, EDSS: Expanded Disability Status Scale, PC-Severity: first principal component extracted from EDSS and MSSS, PC 3dUTPase-EBV: first principal component extracted from IgA, IgM,IgG directed to Epstein-Barr Virus deoxyuridine-triphosphatase (dUTPase-EBV), PC 3dUTPase-HHV-6: first principal component extracted from IgA, IgM,IgG directed to herpesvirus 6 deoxyuridine-triphosphatase (dUTPase-HHV-6), PC 6dUTPases: first principal component extracted from IgA, IgM,IgG directed to both dUTPase-EBV and dUTPase-HHV-6, PC 3EBNA: first principal component extracted from IgA, IgM,IgG directed to Epstein–Barr virus nuclear antigen 1 (EBNA).

**ESF, Table 3. Intercorrelation matrix between between IgG, IgA and IgM against viral antigens and neuropsychiatric rating scales in relapsing-remitting multiple sclerosis (RRMS).**

| Biomarkers | DOI  in RRMS (n=55) | EDSS  in RRMS (n=55) | MSSS  in RRMS (n=55) |
| --- | --- | --- | --- |
| IgG-MBP | 0.219 (0.108) | 0.112 (0.414) | -0.164 (0.230) |
| IgM-MBP | -0.315* (0.019) | 0.214 (0.117) | 0.369** (0.006) |
| IgA-MBP | -0.352** (0.008) | 0.198(0.148) | 0.342* (0.011) |
| IgG-PLP | -0.155 (0.258) | 0.030 (0.826) | 0.197 (0.150) |
| IgM-PLP | -0.186 (0.174) | 0.163 (0.234) | 0.237 (0.082) |
| IgA-PLP | -0.347** (0.010) | 0.120 (0.383) | 0.320* (0.017) |
| IgG-MOG-35-55 | 0.098 (0.477) | -0.088 (0.523) | 0.016 (0.906) |
| IgM-MOG-35-55 | -0.170 (0.216) | 0.244 (0.073) | 0.235 (0.084) |
| IgA-MOG-35-55 | -0.396** (.003) | 0.268* (0.048) | 0.416** (0.002) |
| IgG-MOG-31-55 | 0.098 (0.477) | -0.009 (0.948) | -0.048 (0.726) |
| IgM-MOG-31-55 | -0.277* (0.041) | 0.251 (0.065) | 0.343* (0.010) |
| IgA-MOG-31-55 | -0.433** (0.001) | 0.247 (0.069) | 0.430** (0.001) |
| IgG-CIT-MOG | 0.122 (0.375) | -0.142 (0.300) | -0.050 (0.715) |
| IgM-CIT-MOG | -0.137 (0.318) | 0.161 (0.241) | 0.149 (0.279) |
| IgA-CIT-MOG | -0.381** (0.004) | 0.271* (0.045) | 0.402** (0.002) |
| IgG-MAG-37-60 | 0.056 (0.682) | 0.128 (0.350) | -0.062 (0.651) |
| IgM-MAG-37-60 | -0.213 (0.119) | 0.260 (0.055) | 0.258 (0.057) |
| IgA-MAG-37-60 | -0.425** (0.001) | 0.209 (0.126) | 0.364** (0.006) |
| IgG-GLIAL-CAM-370-399 | -0.127 (0.357) | -0.088 (0.525) | 0.141 (0.306) |
| IgM-GLIAL-CAM-370-399 | -0.137 (0.319) | 0.230 (0.091) | 0.159 (0.245) |
| IgA-GLIAL-CAM-370-399 | -0.465** (0.000) | 0.245 (0.071) | 0.453** (0.001) |

Ig: Immunoglobulin, MBP: myelin basic protein, PLP: proteolipid protein complex, MOG-35-55: Myelin Oligodendrocyte Glycoprotein- amino acids 35 to 55 of the protein, MOG-31-55: Myelin Oligodendrocyte Glycoprotein- amino acids 31 to 55 of the protein, CIT-MOG: citrullinated Myelin Oligodendrocyte Glycoprotein, MAG-37-60: Myelin-Associated Glycoprotein-amino acids 37 to 60 of the protein.
